# Mechanical Strength Improvements of Carbon Nanotube Threads through Epoxy Cross-Linking

**DOI:** 10.3390/ma9020068

**Published:** 2016-01-25

**Authors:** Qingyue Yu, Noe T. Alvarez, Peter Miller, Rachit Malik, Mark R. Haase, Mark Schulz, Vesselin Shanov, Xinbao Zhu

**Affiliations:** 1 Department of Chemical Engineering, Nanjing Forestry University, Nanjing 210037, China; qingyueyunj@126.com; 2 Department of Chemical Engineering, Nanjing Polytechnic Institute, Nanjing 210048, China; 3 Department of Biomedical, Chemical and Environmental Engineering, University of Cincinnati, Cincinnati, OH 45221, USA; millepj@mail.uc.edu (P.M.); haasemr@mail.uc.edu (M.R.H.); 4 Department of Mechanical and Materials Engineering, University of Cincinnati, Cincinnati, OH 45221, USA; malikrt@mail.uc.edu (R.M.); schulzmk@ucmail.uc.edu (M.S.)

**Keywords:** carbon nanotube thread, carbon nanotube fiber, high tensile strength, epoxy cross-linking, 4,4-methylenebis (N,N-diglycidylaniline)

## Abstract

Individual Carbon Nanotubes (CNTs) have a great mechanical strength that needs to be transferred into macroscopic fiber assemblies. One approach to improve the mechanical strength of the CNT assemblies is by creating covalent bonding among their individual CNT building blocks. Chemical cross-linking of multiwall CNTs (MWCNTs) within the fiber has significantly improved the strength of MWCNT thread. Results reported in this work show that the cross-linked thread had a tensile strength six times greater than the strength of its control counterpart, a pristine MWCNT thread (1192 MPa and 194 MPa, respectively). Additionally, electrical conductivity changes were observed, revealing 2123.40 S·cm^−1^ for cross-linked thread, and 3984.26 S·cm^−1^ for pristine CNT thread. Characterization suggests that the obtained high tensile strength is due to the cross-linking reaction of amine groups from ethylenediamine plasma-functionalized CNT with the epoxy groups of the cross-linking agent, 4,4-methylenebis(N,N-diglycidylaniline).

## 1. Introduction

Vertically-aligned carbon nanotubes (CNTs) are one-dimensional nanomaterials with extremely high tensile strength [1,2], elastic modulus [1,3], large aspect ratio, low density, good chemical and environmental stability [4], and high thermal and electrical conductivity [5]. These properties make CNT very attractive for many structural applications, such as aerospace structures, body armor, and sporting goods [6]. However, methods that take full advantage of the excellent mechanical properties of microscale CNT remain elusive. Early studies of CNT-reinforced nanocomposites showed that CNT was an effective reinforcing phase for enhancing the mechanical properties of polymer matrices [7], but the extent of reinforcement was limited by the quality of dispersion, CNT alignment, and load-transfer efficiency between the CNT and the matrix. Thus, it seems that the full potential of CNTs have not yet been utilized in CNT assemblies like fibers, threads, and composites.

CNTs are chemically stable to large variety of environments; therefore, a poor interaction between the CNTs and their matrix is common in most applications where CNTs are used as reinforcing additives. Reactive sites known as functional groups need to be incorporated in order to improve their interaction with the medium. There are multiple CNT functionalization methods, most of them are chemical [8,9,10,11]. A very effective method is plasma functionalization, which modifies the surface morphology of CNTs by introducing defects and functional groups in the CNT surface [12]. Plasma has been used extensively for surface modification of multiple nanomaterials, and it has proven to be quite effective to improve dispersions and breaking up nanoparticle agglomerations [13,14,15,16,17,18,19]. Utilizing plasma methods to treat or modify carbon nanomaterials can widen the range of compatible polymer matrices. Tailoring CNT functional groups it is possible to increase their dispersion homogeneity within polymers, enhance the interfacial bonding to the polymer matrix, and achieve the desired properties of the resulting polymer nanocomposites [13].

Composite materials based on epoxy resins are widely used for applications where a high modulus, thermal stability, and solvent resistance are required [20]. Glycidyl amine epoxy resins are used as low temperature curing structural epoxy composite materials [21]. 4,4-methylenebis(N,N-diglycidylaniline) (MBDGA), a kind of glycidyl amine epoxy resin studied in this report, has four glycidyl epoxy groups in a molecular structure. Hypothetically, that means a molecule of MBDGA can react with four amine groups cross-linking one or more functional groups in the CNTs. After the reaction is complete, we expect that the tensile strength of CNT yarn functionalized with glycidyl amine resin will be much higher than pristine CNT thread. Although the cross-linking approach may be applicable to all CNTs, this manuscript is focused on MWCNTs.

CNT fibers and threads, terms used indistinctly in the literature, are forms of CNT assemblies that have shown performances below individual CNTs. Regardless of their assembly method, individual CNTs within the fiber are held together by van der Waals forces only. Covalent bond formation between CNTs is an approach that have potential to increase the mechanical strength of the assembly [22,23], as well as improve other physical properties [24]. Multiple cross-linking chemistries have been attempted but few of them have been focused on the mechanical strength improvements [22,23,25,26,27]; therefore, the search for an ideal reaction that will crosslink CNTs within the fiber continues. Here, we report a six-fold increase of the mechanical strength of the CNT threads after covalent cross-linking. Our strategy is to use free radical and ionic species in helium/nitrogen plasma in order to create active sites on CNT surface for bonding with ethylenediamine (EDA) molecules, thus enabling amine functionalization of the nanotubes. These amine functional groups will reacts with an epoxy based cross linker known as MBDGA.

## 2. Experimental

### 2.1. Sample Preparation

MWCNTs with outer diameters ranging 6–20 nm, wall number 2–10, and length 400–500 µm were manufactured from Nanoworld Laboratories of University of Cincinnati by a water-assisted CVD method [28,29,30,31]. MWCNT threads were assembled from drawable MWCNT arrays using the dry spinning method which starts with the synthesis of vertically-aligned drawable MWCNTs, typically between 400 and 500 µm in length. The process starts with catalyst thin film deposition of Fe/Co alloys (1.2 nm) on 4-inch Si wafers that has 5 nm Al_2_O_3_ layers deposited by sputtering (Kurt J. Lesker, Jefferson Hills, AL, USA) as a buffer layer. The Si wafer substrates were scribed and broken into 2-inch long and variable width (up to 1.5 inch) substrates that were loaded into a commercial CVD reactor ET3000 from CVD Equipment Corporation (Central Islip, NY, USA). The pressure during the growth process is 740 Torr. The reactor is heated to 400 °C for annealing under Ar, after 2 min the reactor is ramped to 750 °C, and a 300 sccm mixture of C_2_H_4_ and 1000 sccm of Ar are introduced at this temperature for 20 min. Upon growth completion, 30 sccm of H_2_O and 2000 sccm of Ar were delivered during cooling to promote CNT array detachment.

Drawable MWCNT arrays are ideal to make different diameter of MWCNT threads via simultaneous twisting and winding. A Surfx Atomflo 400D plasma system was used to create atmospheric pressure plasma from a mixture of helium and nitrogen gases (30:0.4 L/min) operating at 80 W (gases are ultra-high purity, from Wright Brothers, Montgomery, AL, USA). EDA in vapor form was injected into the plasma jet via flow of helium a t 0.5 L/min through liquid EDA.

After toluene densification, MWCNT thread was functionalized with EDA, and pristine thread was kept unfunctionalized as a control. The functionalized MWCNT thread was then added to a 5 wt % MBDGA (Sigma-Aldrich, St. Louis, MO, USA) toluene solution and allowed to react at room temperature for 12 h. After the cross-linking reaction, the thread was washed with toluene to remove the polymer excess at its surface. The mass of liquid curing agent (LCA), EDA, can be calculated by using the follow equation:

$$M_{LCA}=\frac{M\times G}{H_{n}}$$

*M_LCA_*—mass of liquid curing agent;
*M*—molecular weight of LCA;
*G*—epoxide number of glycidyl amine epoxy;
*H_n_*—number of active hydrogen.

The EDA/MBDGA cross-linked thread was dried in an oven at 120 °C for 30 min to vaporize the solvent.

### 2.2. Characterization

The surface morphology and diameter of CNT thread were observed under a scanning electron microscope (ESEM, Philips XL-30 Field emission, Eindhoven, The Netherlands). CNT thread samples were analyzed by Raman, in a Via micro-Raman spectrometer (Raman, Gluocestershire, UK), X-ray photoelectron spectroscopy (XPS, ESCALAB PHI-5300, Waltham, MA, USA) and Thermogravimetric Analysis and Differential Scanning Calorimeter (TA-DSC, NETZSCH STA-409PC, Burlington, MA, USA) to determine the structural characteristics and chemical elements of the material before and after EDA plasma-functionalization, as well as of MBDGA cross-linked CNT. The diameter of each CNT thread was obtained by observing twenty positions under SEM. When testing the mechanical properties of all thread samples, the active gauge length was 16 mm and a strain rate was of 1.0 mm·min^−1^, at least ten specimens were taken from the same thread and measured to obtain the average tensile strength of the yarn. The tensile strength-extension curves were recorded by an INSTRON 5948 Micro Tester equipped (Norwood, LA, USA) with a 5N load cell. Thermogravimetric analysis took place under air flow at a rate of 30 mL·min^−1^ and heating rate of 5 °C·min^−1^.

## 3. Results and Discussion

### 3.1. Mechanical Properties and Chemical Reaction Mechanism

**Figure 1:**
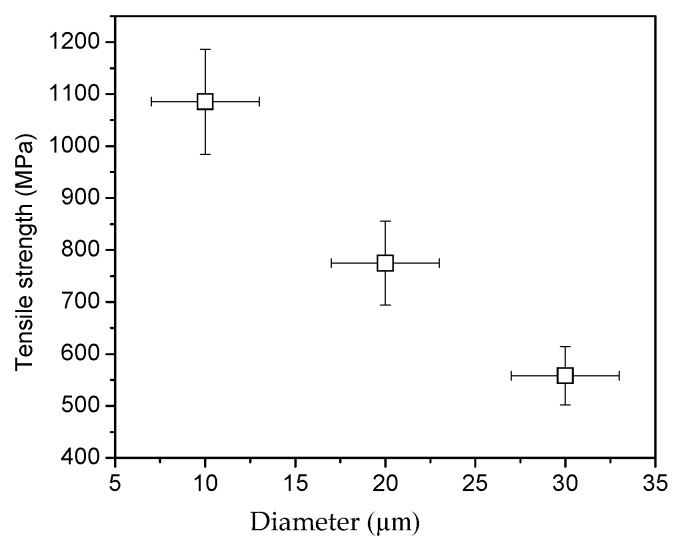
Tensile Strength of different diameter EDA/MBDGA cross-linked MWCNT threads.

**Figure 2:**
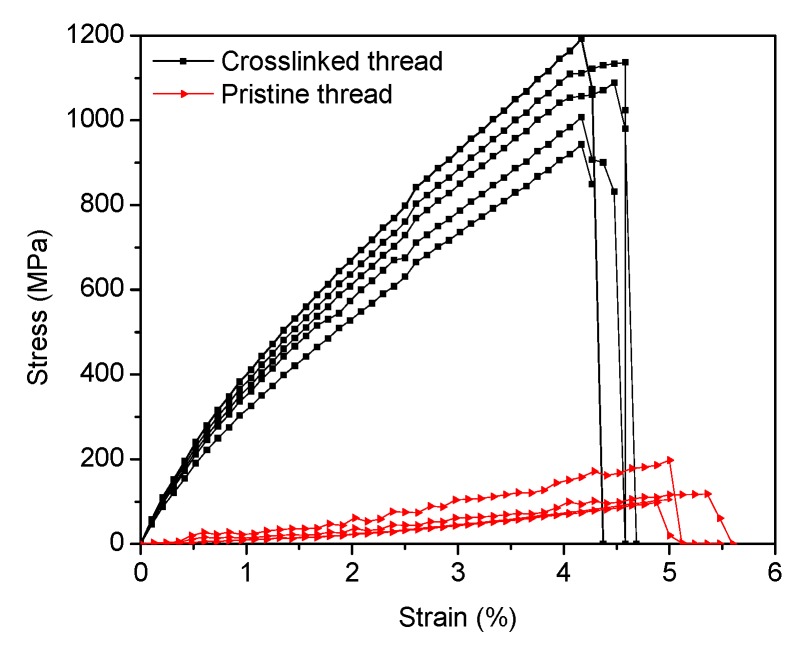
Tensile strength-extension curve of pristine MWCNT thread compared to EDA/MBDGA cross-linked MWCNT thread.

CNTs within a dry spun thread are held together by simple van der Waals bonding only. Slipping between CNTs is the most predominant form of failure. Covalent bonding, a much stronger type of chemical bonding than van der Waals, is highly desirable and has already shown improvements of the thread strength by bridging CNTs within the thread. However, to make meaningful improvements in strength, the number of covalent bonds within the thread has to increase which can bring significant challenges. One of these challenges is the ability to create a high number of functional groups and effectively introduce the cross linker with the thread. Due to the large number of CNTs within a thread, (10^6^, approximately) [32] and the initial bundle density, effective penetration of the thread by the cross-linker will be limited unless thread diameter is significantly reduced. A proof of this hypothesis is supported by thread strength dependence from the diameter among the cross-linked threads. For diameters above 10 µm, the tensile strength of the EDA/MBDGA cross-linked threads decreased with increasing CNT thread diameter as shown Figure 1. We speculate that this is due to the number of amine groups we create by EDA plasma within the thread, and the cross-linking agent penetration depth in the thread. Plasma is an effective surface functionalization technique, however plasma functionalization may decay radially in a cylindrical object such as the CNT thread. Thus, the number of functional groups within the thread will decay from surface to the center; therefore, a more effective functionalization may be accomplished on small diameter CNT threads. The solvent (cross-linker) penetration should also be more effective when infiltrating small diameter CNT thread. Figure 2 shows the tensile stress-strain curves of pristine CNT thread and EDA/MBDGA cross-linked CNT threads. The starting threads named as “pristine thread” have an average tensile strength of 194 MPa and extension at failure of about 5%. After treatment with toluene, functionalized with EDA plasma, and cross-linked with MBDGA the tensile strength of MBDGA cross-linked CNT yarn increases to 1192 MPa and extension at failure of 4.2%.

**Figure 3:**
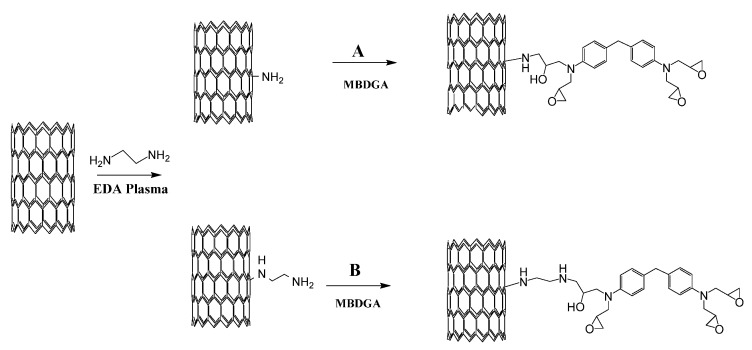
Proposed cross-linking reaction mechanism of EDA functionalized MWCNTs in threads and fibers. Epoxy functional groups in MBDGA are the actives sites that promote covalent bond formation between MWCNTs.

The tensile strength of MBDGA cross-linked CNT thread shows a drastic improvement when compared to pristine CNT thread, as shown Figure 2. These results support the idea of covalent bond formation between CNTs within the thread, because neither the polymer itself nor the solvent densification could achieve such improvement alone. To understand and explain our results, we propose a chemical reaction mechanism shown in Figure 3. As shown, CNTs are functionalized by reactive chemical amine species generated by the plasma; therefore, it is reasonable to think that EDA plasma will generate two types of functional groups: with one and two N in the reactive amine group site. These intermediate products will react with one of the epoxy groups on the MBDGA following the reaction paths: A and B (Figure 3). We think the cross-linking effectiveness of MBDGA is high because each of the byproducts generated in step A and B, have a potential to react with additional three amines groups in the same or neighboring CNTs. Ideally, the MBDGA should bridge up to four CNTs within the tread if the arrangement and position allows.

### 3.2. The Effects of Defects and Functional Groups

#### 3.2.1. SEM Analysis

**Figure 4:**
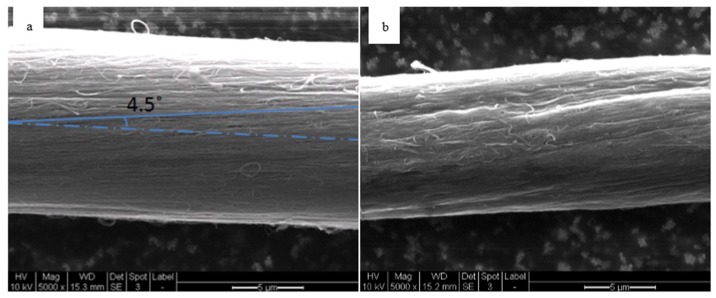
SEM images of (**a**) pristine; and (**b**) EDA/MBDGA cross-linked MWCNT.

An encouraging fact is that CNT thread after covalent cross-linking shows a smaller diameter than its pristine counterpart, suggesting that density of CNTs increased within the thread by reducing the distance between them. SEM images shown in Figure 4a,b support this observation. Typically pristine CNT threads from drawable CNT arrays have low density, which increase as they are compressed or exposed to organic solvents. The threads are assembled through simultaneous twisting and drawing CNT bundles from vertically-aligned MWCNTs, they obtain their cylindrical shape due to the continuous twisting. The tested thread samples in our studies had a uniform diameter of 13 µm, as shown in Figure 4a. After EDA plasma-functionalization, MBDGA cross-linking, and toluene washing the thread retains a uniform diameter, though the diameter was reduced to 9 µm, as shown in Figure 4b.

#### 3.2.2. Raman Analysis

**Figure 5:**
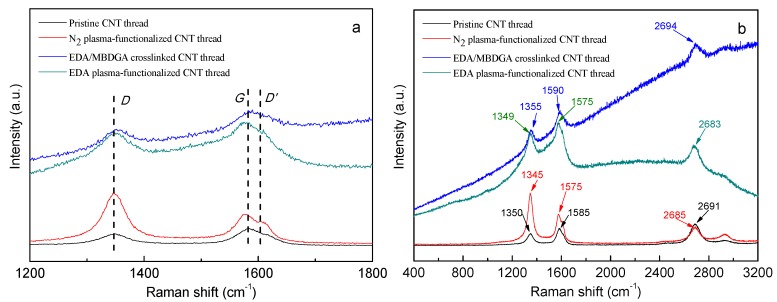
Raman spectra (514 nm) of pristine, plasma functionalized, EDA/MBDGA cross-linked MWCNTs threads; (**a**) shows Raman shifts of main peaks D, G, and D’ at pristine state and after functionalization of MWCNTs; (**b**) larger Raman shift window visualizing Raman shifts for each peak and the shift of G peak after functionalization.

The continuous generation of highly reactive species generated in the plasma environment, and subsequent collision with MWCNTs is a simple approach to chemical functionalization. Plasma ions are known for their ability to modify surface chemistry of materials which is supported by its Raman spectra (514 nm) that shows an increase of *I*_D_/*I*_G_ ratios (from 0.69 to 1.68) for functionalized MWCNTs compared to their pristine counterparts, Figure 5a. Main peaks D, G, and D’, as well as the Raman peaks shifting after functionalization are visualized. Furthermore, D’ peak generally associated with the disorder on CNTs shows a relative intensity increase; D’ that is barely visible in the pristine MWCNTs becomes a quite evident peak after functionalization. Figure 5b shows the positions of the peaks and their correspondent Raman shifts of the main peaks. As much as 10 cm^−1^ shifting in the G peak is observed for amine-functionalized compared to pristine MWCNTs. Figure 5b also suggests that current plasma treatment does produce moderate damage to the CNTs, because similar Raman spectra have been reported for plasma-functionalized and pristine CNTs [33]. Fortunately this defects introduced by plasma treatment into CNT surface, are reactive sites that are used for replacing by amine functional groups. To increase the amount of amine groups, nitrogen plasma is used to functionalize CNTs.

#### 3.2.3. XPS Analysis

**Figure 6:**
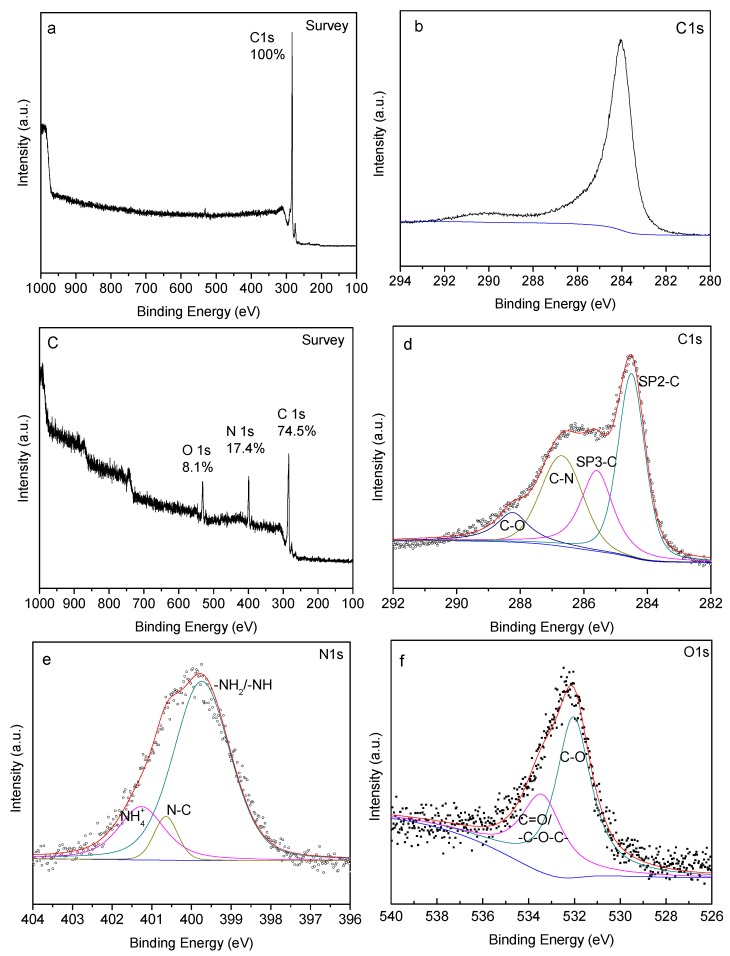
XP spectra of MWCNTs, (**a**) pristine survey; (**b**) pristine C1s region and EDA plasma-functionalized CNT; (**c**) survey; (**d**) C1s region; (**e**) N1s region; and (**f**) O1s region.

Additional evidence of functionalization is provided by X-ray Photoelectron Spectroscopy (XPS). Surface element composition of functionalized CNTs are analyzed and compared to XPS of pristine CNTs. In the XPS spectra, each peak is fitted by using the binding energy of standard carbon, 284.5 eV [34]. Figure 6a shows and XPS elemental survey of pristine CNTs which shows the presence of elemental carbon only. Figure 6b shows the main peak position at 284.5 eV is for pure *sp*^2^ hybridized CNTs and the peak position at 290.5 eV is for characteristic shakeup line of carbon in aromatic compounds (π-π* transition) [34,35]. After functionalization with EDA plasma, the nitrogen amount on the CNT surface reaches 17.4% as shown in the XPS survey Figure 6c. The presence of N suggests EDA functionalization of the CNTs at the CNT thread surface. The asymmetric peak at 284.5 eV is assigned to *sp*^2^-hybridized graphitic carbon atoms and *sp*^2^ carbon atoms bound to hydrogen. Figure 6d shows C1s region of the XPS spectra on EDA plasma functionalized CNTs. In Figure 6d shows the carbon nitrogen (C–N) bond peak at 286.73 eV and carbon oxygen (C–O) bond peak at 288.25 eV. C–N bond can be observed after EDA plasma functionalization. The peak at 288.4 eV in Figure 6d, is a typical peak of carbon atoms bounded to oxygen atoms by single bonds caused by high temperature oxidation of CNTs. In Figure 6d, the peak at 285.59 eV originates from *sp*^3^-hybridized carbon atoms which also shows that defect are introduced onto the CNT surface [35,36]. The peak at 399.72 eV in Figure 6e comes from the amines group on EDA plasma-functionalized CNTs, while the peaks appearing at 400.62 eV and 401.28 eV are from the N–C bond and NH_4_^+^ ion on EDA plasma-functionalized CNTs [35,36]. The peaks in Figure 6f appearing at 532.01 eV and 533.49 eV represent C–O and C=O respectively from high temperature plasma oxidation [37]. The oxygen amount on the CNT surface reaches 8.1%, shown in Figure 6c, after functionalized with EDA plasma.

**Figure 7:**
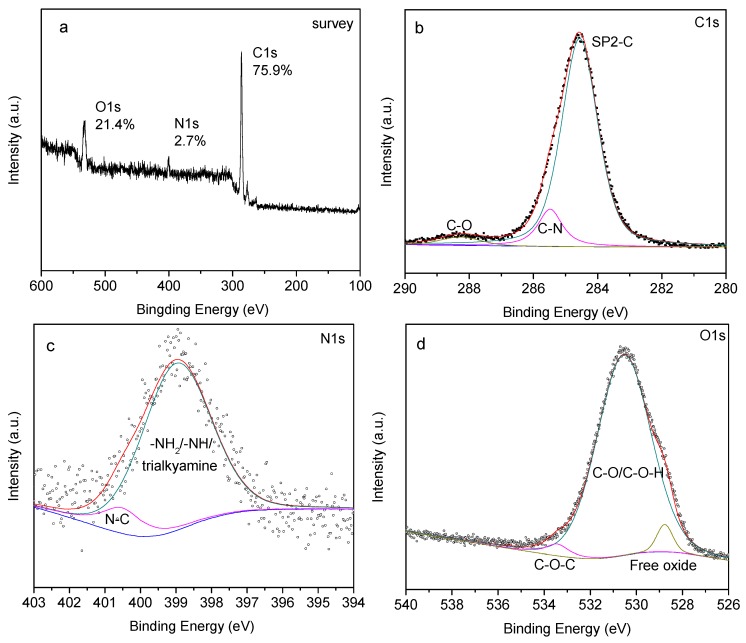
XP spectra of EDA/MBDGA cross-linked MWCNTs (**a**) survey; (**b**) C1s region; (**c**) N1s region; and (**d**) O1s region.

XPS of EDA/MBDGA cross-linked CNTs are shown in Figure 7. A Comparison of nitrogen and oxygen mass percentage of EDA plasma-functionalized CNTs (Figure 6c) and the EDA/MBDGA cross-linked CNTs (Figure 7a) shows that nitrogen mass decreasing from 17.4% to 2.7%, and the oxygen mass increase from 8.1% to 21.4%. This increase in mass percentage of N and O on the XPS spectrum suggests that amine groups in the EDA plasma functionalized have reacted with epoxy groups present in MBDGA. The C1s region XPS spectra (Figure 7b) shows the peak position at 284.5 eV is for *sp*^2^ hybridized CNTs, the C–N bond peak at 285.47 eV and the C–O bond peak at 288.36 eV. The small shift of the peak position compared with EDA plasma-functionalized CNTs (Figure 6d) can support the cross-linking reaction. In Figure 7c, the peak at 398.94 eV mainly comes from the trialkylamine group that is formed after the reaction of amine groups on EDA plasma-functionalized CNT with epoxy groups present in MBDGA [36,37,38]. The peaks in Figure 7d appear at 530.50 eV and 533.45 eV are from the C–OH for open-ring reaction of epoxy groups present in the cross-linker with amine groups of the EDA plasma-functionalized CNTs and the C–O–C for open-ring reaction of epoxy groups in MBDGA with another epoxy group respectively during the cross-linking reaction [36]. A peak at 528.80 eV comes from free oxygen on the surface of EDA/MBDGA cross-linked CNT. The XPS spectra are consistent with the chemical reaction mechanism shown in Figure 3. The robust covalent bonds formed between the amino groups of functionalized MWNTs and epoxy matrix can provide stronger interfacial shear stress, thereby improving the mechanical properties of the epoxy matrix [39].

#### 3.2.4. Thermogravimetric Analysis

**Figure 8:**
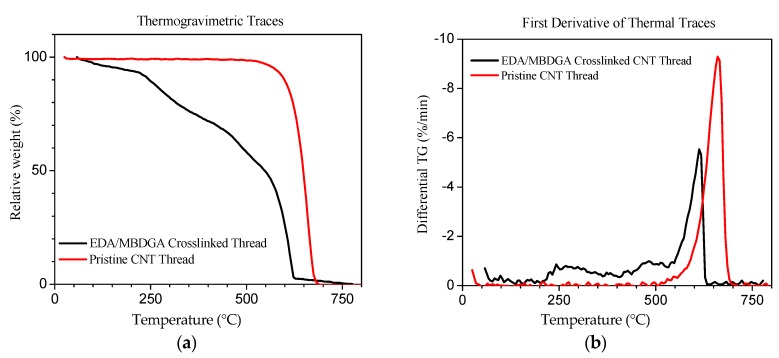
TGA curves of pristine MWCNT thread and EDA/MBDGA cross-linked MWCNT thread. (**a**)Thermogravimetric traces; (**b**) First derivative of thermal traces.

TGA is carried out in air for the CNTs samples from 50 °C to 750 °C (Figure 8). Pristine CNTs remain fairly stable up to 500 °C, after which they begin to oxidize rapidly. Peak oxidization occurs just below 700 °C, and takes place across a single, well defined peak Figure 8a. The plasma-treated and cross-linked thread, in contrast, does not remain stable during heating; there is a steady mass loss from the onset of heating, which increases rapidly towards the end of the heating cycle. The mass loss at the outset is likely to be volatiles, perhaps residual solvent; this material is driven off by 200 °C. There are several cusps in the subsequent portions of the TGA trace, but the first derivative is unable to resolve them into clear peaks, Figure 8b. These are probably the polymer crosslinks decomposing but the limited polymer concentration may contribute to achieve well defined peaks. The last part of the TGA trace, starting around 500–550 °C, shows a precipitous decline; the first derivative shows a clear peak at about 600 °C. This last region most likely corresponds to the CNT materials. Comparing the range of the first derivative peak with the mass loss in the thermogravimetric trace, it seems that CNTs constitute about 60% the material by mass. These CNTs have more defects than pristine material, as evidenced by the lower oxidation peak.

Another important property of CNT threads is their electrical conductivity. Comparison of conductivities between pristine and EDA/MBDGA cross-linked CNT threads are shown in Table 1. Electrical conductivity of pristine CNTs is 3984.26 S·cm^−1^ is higher than of cross-linked CNTs, 2123.40 S·cm^−1^. However, among the cross-linked MWCNTs it appear that larger diameter threads are more conducting than small diameter ones, suggesting that plasma functionalization may be more effective for small diameter threads. We think that further studies are needed to explain this reduction in conductivity after functionalization, although it seems reasonable to think that presence of plasma damaged CNTs as well as the presence of extra polymer within the thread will effectively reduce the electrical conductivity of the thread.

**Table 1:** Electrical conductivity of MWCNT threads.

Sample	Conductivity (S·cm^−1^)	Standard Deviation (S·cm^−1^)
Pristine CNT thread	3984.26	110.67
EDA/MBDGA cross-linked CNT thread	2123.40	86.16

## 4. Conclusions

Mechanical and electrical properties of pristine CNT thread and EDA/MBDGA cross-linked CNT thread has been explored. Significant mechanical strength improvement has been observed in the cross-linked CNT threads. Pristine CNT thread and EDA/MBDGA cross-linked CNT threads have a tensile strength of 194 and 1192 MPa and electrical conductivities of 3984.26 and 2123.40 S·cm^−1^, respectively. Raman spectroscopy supports successful functionalization of CNTs on the threads. The conducted XPS study supports EDA plasma functionalization of the thread conforming CNTs. XPS also supports the cross-linking, and quantified the nitrogen amount on the surface of the thread, which reached 17.4% for EDA plasma-functionalized CNTs and 2.7% for MBDGA cross-linked CNTs. The above results support that amine groups on the CNT surface reacted with epoxy group present in the cross linker MBDGA. TGA revealed that EDA/MBDGA cross-linked CNT thread has good thermal stability, though lower than CNTs. We think that the approach described in this work is a promising effort for bridging that gap between the extraordinary mechanical properties of individual CNTs and those of CNT assemblages such as threads and sheets.
